# Case Report: An unusual case of a transposition of the great arteries with a double aortic arch: a highly complex fetal diagnosis with an unpredictable outcome

**DOI:** 10.3389/fcvm.2024.1351530

**Published:** 2024-04-12

**Authors:** M. Masci, A. Missineo, C. M. Campanale, P. Moras, M. C. Colucci, L. Pasquini, A. Toscano

**Affiliations:** ^1^ Perinatal Cardiology Unit, Bambino Gesù Children’s Hospital IRCCS, Rome, Italy; ^2^ Department of Maternal-Fetal Medicine, Catholic University of the Sacred Heart, Rome, Italy

**Keywords:** fetal diagnosis, transposition of the great arteries, double aortic arch, vascular ring, fetal echocardiography

## Abstract

Published data estimate the prevalence of the vascular ring at approximately 7 per 10,000 live births. The association of a double aortic arch with a D-transposition of the great arteries has been rarely described in the literature. In this study, we report the prenatal diagnosis of a 28-year-old woman. A fetal echocardiography at a gestational age of 24 weeks + 6 days showed a D-transposition of the great arteries and a double aortic arch with a ventricular septal defect and pulmonary stenosis. On the first night after birth, the baby experienced an increase in lactate levels, with the rate of oxygen saturation consistently below 80%. A few hours after birth, the patient underwent a Rashkind procedure. An echocardiography, CT chest x-ray, and CT angiogram confirmed a diagnosis with a severe reduction of the tracheal lumen (>85%) and bronchomalacia. Then, the patient underwent posterior tracheopexy and aortopexy and later an arterial switch operation, ventricular septal defect closure, and resection of a part of the infundibular septum, accepting the risk of potential neoaortic obstruction. The literature has reported only two cases of patients with a fetal echocardiogram diagnosis. Therefore, our patient is only the third one with a fetal diagnosis and the second one with a complex intracardiac anatomy, characterized not only by a ventricular septal defect but also by two separate components of the obstruction (a bicuspid valve and a dysplastic valve with a posterior deviation of the infundibular septum). In conclusion, a D-transposition of the great arteries with a double aortic arch remains an extremely unusual association. The clinical outcome of these patients presents a high degree of variability and is entirely unpredictable in prenatal life. Our greatest aim as fetal and perinatal cardiologists is to improve the management and outcome of these patients through a fetal diagnosis, recognizing types of congenital heart disease in newborns who require early neonatal invasive procedures.

## Case description

**Figure 1:**
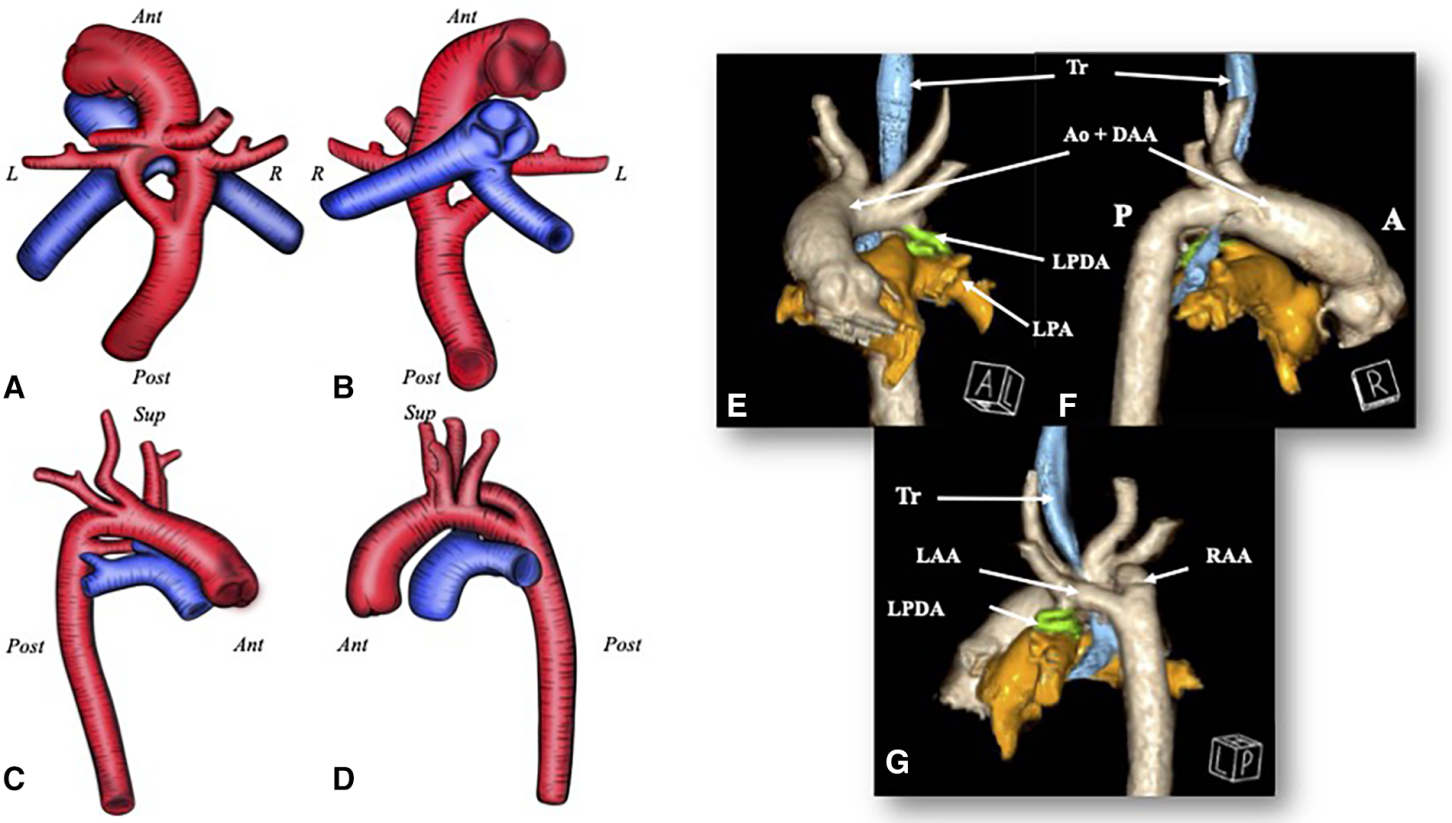
A D-TGA with a DAA. Image (**A–D)** The red blood vessel is the aorta with a double aortic arch; the blue one is the pulmonary artery. (**A**) A view from above; (**B**) a view from below; (**C**) a view from the right side; and (**D**) a view from the left side. (**E–G**) A CT scan 3D rendering of the double aortic arch in the transposition of the great arteries and the relationship with the trachea. (**E**) An anterior view; (**F**) a view from the right side; and (**G**) a posteroleft view. Ant, anterior; Post, posterior; R, right; L, left; Ao, aorta; LPA, left pulmonary artery; LPDA, left patent ductus arteriosus; Tr, trachea.

**Figure 2:**
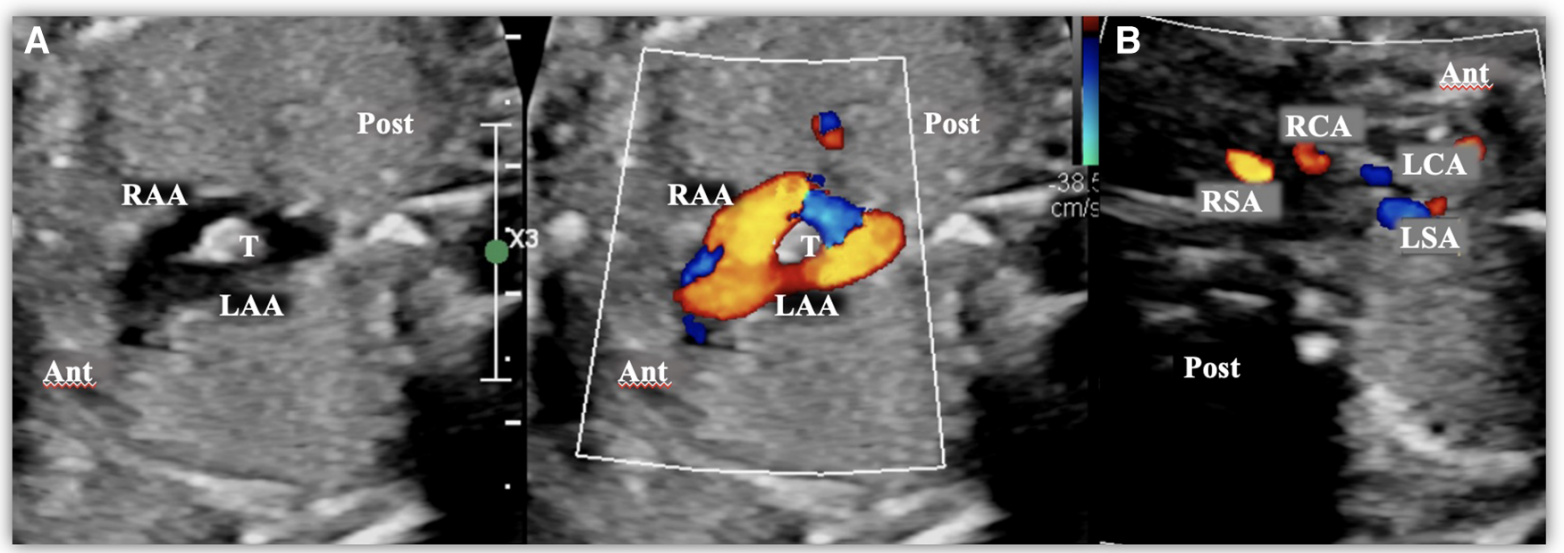
Images taken from a fetal transthoracic echocardiogram. (**A**) A cross-sectional view at the level of the aortic arch, with a color Doppler, where it is possible to observe the complete vascular ring surrounding the trachea, consisting of a double aortic arch. (**B**) A cross-sectional view at the level of the epiaortic vessels, with a color Doppler, showing the four epiaortic vessels originating separately (two on the right and two on the left). Ant, anterior; LAA, left aortic arch; LCA, Left carotid artery; LSA, left subclavian artery; Post, posterior; RCA, right carotid artery; RSA, right subclavian artery; T, trachea.

**Figure 3:**
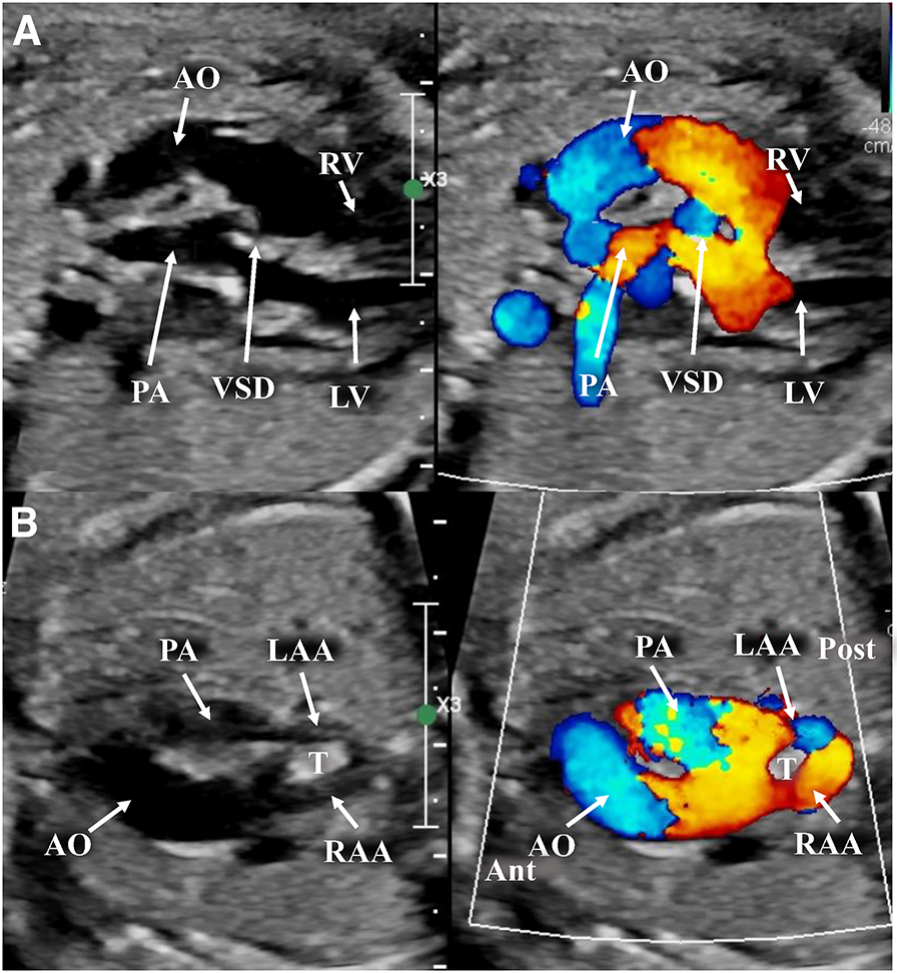
Images taken from a fetal transthoracic echocardiogram. (**A**) A long-axis view, with a color Doppler, where it is possible to observe the D-transposition with the anterior aorta and posterior pulmonary artery; the ventricular septal defect, with a dysplastic pulmonary valve. (**B**) An off-axis view at the level of the aortic arch, with a color Doppler, showing the anterior aorta with its complete vascular ring surrounding the trachea, consisting of a double aortic arch, and the posterior pulmonary artery. Ant, anterior; LAA, left aortic arch; LV, left ventricle; Post, posterior; RV, right ventricle; T, trachea.

In this study, we report the prenatal diagnosis of a combined D-transposition of the great arteries (D-TGA) with a ventricular septal defect (VSD), pulmonary stenosis, and a double aortic arch (DAA) in a 28-year-old woman at 24 weeks and 6 days of gestational age (GA). No family history of congenital heart disease or genetic disorders was found. A fetal echocardiography revealed a normal visceral situs, atrioventricular concordance, and a D-TGA, with the anterior aorta arising from the right ventricle and the posterior pulmonary trunk arising from the left ventricle. Two symmetrical aortic arches surrounded the trachea, each giving rise to separate arteries, respectively: the left carotid and left subclavian arteries, and the right carotid and right subclavian arteries (Figures 1, 2). Subpulmonary VSD and pulmonary valve (PV) dysplasia with a right outflow tract obstruction were also noted (Figure 3).

The baby was born by a scheduled cesarean section (GA 38 weeks + 5 days). At birth, the oxygen saturation value was 75%. Prostaglandin infusion was started after placing an umbilical catheter. Thereafter, the infant was quickly transferred from the birth center to our hospital.

A postnatal echocardiography confirmed a D-TGA with a DAA, with a dominant right-sided aortic arch, a hypoplastic left-sided aortic arch, and a small tortuous left ductus arteriosus (DA). The ductus arteriosus was non-hemodynamically significant. The interatrial communication was not a restricted foramen ovale. The PV was dysplastic and bicuspid with a posterior deviation of the infundibular septum. The degree of obstruction was classified as “moderate with a dynamic component.” The coronary arteries had a normal origin from a pathological perspective, but both were positioned in a paracommissural manner.

After a few hours, the patient developed severe acidosis, hypercapnia, dyspnea, and respiratory distress and required intubation. On the first night after birth, the baby experienced an increase in lactate levels, with the oxygen saturation rate consistently below 80%. Therefore, in order to allow an efficient mixing between the two circulations, atrial septostomy, as laid down by American cardiologist William Rashkind, was performed. After adequate mixing was achieved at the atrial level, we noticed an improvement in the oxygen saturation rate (>85%) and a gradual decrease in lactate levels, following which prostaglandin infusion was initiated.

A CT chest x-ray with angiography revealed severe tracheal narrowing with subocclusion (>85%) and tracheobronchomalacia caused by a vascular ring (VR), secondary to compression (Figure 1). Fibrobronchoscopy was performed the day before the surgery. The procedure, which was conducted with the patient awake under sedation but spontaneous breathing, revealed an approximately 70% reduction in the size of the respiratory tract at the transition between the middle and the distal third of the trachea.

The patient was admitted in the pediatric intensive care unit (ICU) but developed multiple respiratory infections caused by *Klebsiella pneumoniae*, *Enterobacter cloacae*, and *Acinetobacter baumannii*, which complicated the respiratory condition. Furthermore, multiple extubation attempts failed. The prolonged hospitalization, the tracheomalacia, and recurrent infections had certainly worsened the respiratory condition. For this reason, at seven days of life, the hypoplastic left arch and DA were resected to relieve the severe tracheobronchomalacia and its consequences. Despite this, extubation was unsuccessful, probably because of the unfavorable anatomy related to the TGA causing vascular compression.

Furthermore, at 2 months and 5 days of life, the patient underwent posterior tracheopexy and aortopexy with extracorporeal circulation. The cardiac surgeon secured a complete release of the esophagus, trachea, and left transverse arch by dissecting the surrounding tissue. Intraoperative endoscopic control and a 1-month follow-up bronchoscopy were performed, documenting a favorable caliber of the trachea and bronchi. Nevertheless, the patient remained dependent on invasive mechanical ventilation. Unfortunately, immediately after the procedure, a brain ultrasound revealed a slight and blurred subcortical hyperechogenicity in the left frontoparietal region, without an evident cavitation area. This was an element probably related to ischemic lesions. Consequently, a brain CT revealed two areas of cortico-subcortical hypodensity in the frontal and temporal regions on the right, indicative of new brain ischemic lesions. Given the timing of the onset, these lesions appeared to be correlated with extracorporeal circulation.

**Figure 4:**
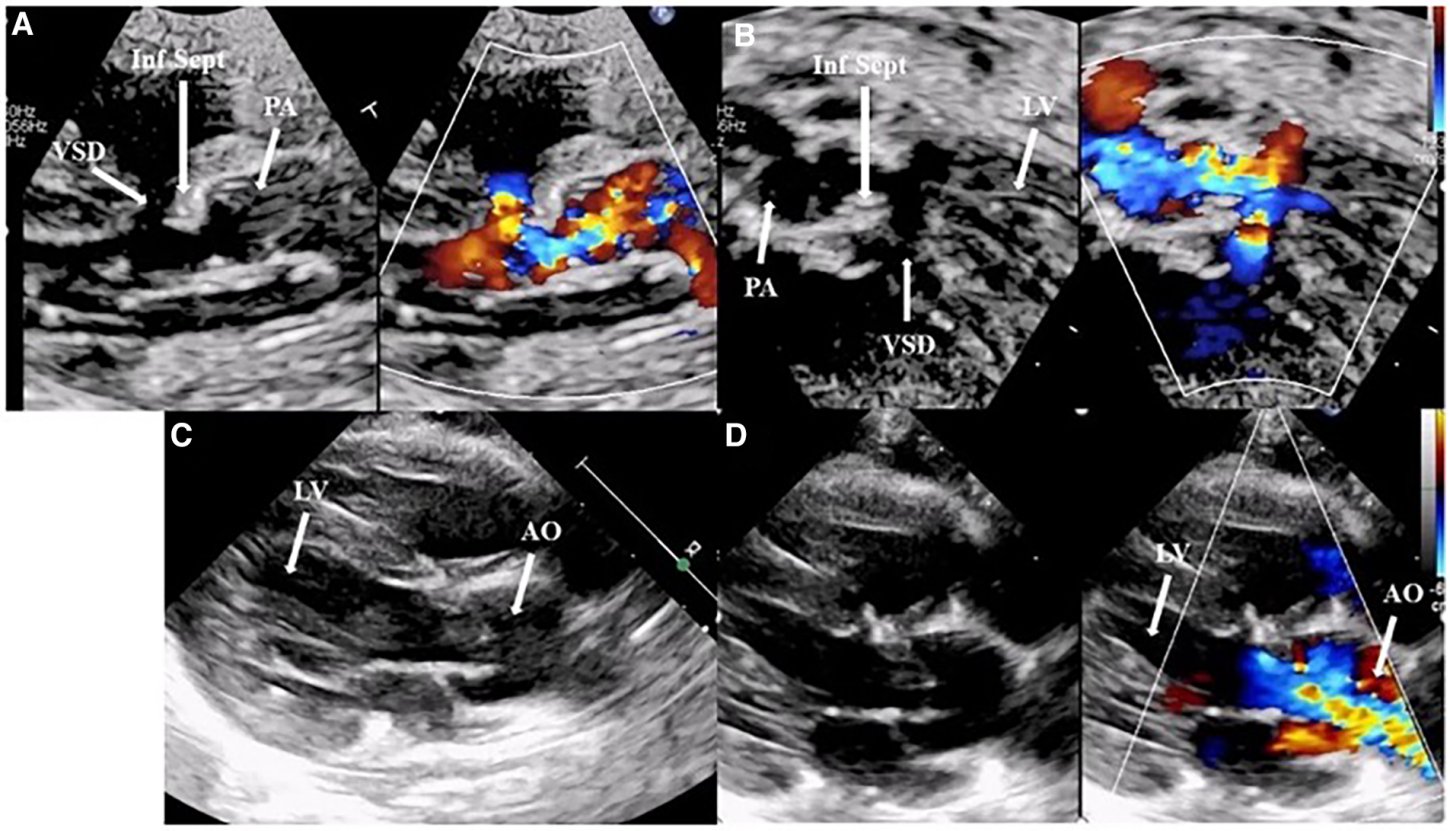
(**A,B**) A preoperative transthoracic echocardiogram: a parasternal long-axis view and five-chamber view with and without a color Doppler, highlighting the posterior deviation of the infundibular septum causing subvalvular and valvar pulmonary obstruction and the restrictive interventricular septal defect. Images (**C,D**), A postoperative transthoracic echocardiogram: a parasternal long-axis view without and with a color Doppler, showing an excellent surgical outcome after an arterial switch operation, closure of the interventricular defect, and resection of the infundibular septum. AO, aorta; Inf Sept, infundibular septum; LV, left ventricle; PA, pulmonary artery.

Because of the restrictiveness of the foramen ovale and the poor clinical improvement, a second Rashkind procedure was performed with less improvement in clinical conditions. During hospitalization, the echocardiographic assessment of the VSD highlighted an evolution toward restrictiveness. After addressing the respiratory complications, the patient, who weighed 3.5 kg, underwent an arterial switch operation (ASO), VSD closure, and resection of a part of the infundibular septum, accepting the risk of potential neoaortic obstruction (Figure 4). During the intraoperative inspection, the neoaortic valve was bicuspid with normal leaflet excursion. The leaflets appeared slightly thickened, but the ring seemed to be of adequate size. The decision to perform an ASO instead of the Nikaidoh or Rastelli procedure was based on the patient's low weight. After the patient achieved clinical stability, he was discharged.

At the 1-year follow-up (6 months after corrective surgery), the patient was found to be stable, despite having a severe impairment of neurological function due to ischemic–hemorrhagic lesions; this was followed up with a brain MRI. He developed a mild neoaortic insufficiency with a mean pressure gradient of 10 mmHg. The patient is currently undergoing regular cardiological and neurological follow-ups.

## Discussion

Our patient represents a clinical case of a prenatal diagnosis of a D-TGA, VSD, and pulmonary and subpulmonary stenosis associated with a DAA, diagnosed in fetal life and confirmed during the postnatal period.

Published data estimate the prevalence of a VR at about 7 per 10,000 live births (1). The incidence of the DAA in the literature is 1.5 per 10,000. The most common type of vascular arch is the right aortic arch (RAA) with an aberrant left subclavian artery with an incidence of 5.4 per 10,000 (2).

The DAA is attributed to the absence of regression of the right fourth branchial arch during embryogenesis.

The VR can be isolated or associated with intracardiac and/or extracardiac anomalies. With regard to the DAA, an underlying cardiac diagnosis is prevalent in up to 12% of cases and includes a VSD, Tetralogy of Fallot, and other complex congenital cardiac conditions (3).

The association of a DAA with a D-TGA has been rarely described (Table 1) in the literature, with only nine cases of patients being described to date. Of these nine patients, six were detected by a postnatal diagnosis with echocardiography (4), angiocardiography (5, 6), direct intraoperative inspection (7), or necropsy (8). The literature has reported just two cases of patients with a fetal echocardiogram diagnosis (9, 10). Therefore, our patient is the third one with a fetal diagnosis and the second one with a complex intracardiac anatomy, characterized not only by a VSD but also by two separate components of the obstruction (a bicuspid valve and a dysplastic valve with a posterior deviation of the infundibular septum).

**Table 1:** Patients with a D-transposition of the great arteries with a double aortic arch described in the literature.

Source	Age/sex	Anatomy	Diagnostic method	Surgery	Comments
Von Siebold (12)	Newborn/—	D-TGA, DAA	Necropsy		
Higashino and Ruttenberg (8)	11 days/girl	D-TGA-DAA	Necropsy	Atrial septectomy	Death 10 h after surgery due to respiratory distress
Kupferschmid (13)	1 day/girl	D-TGA, DAA, VSD, PDA (no side description), PFO	Angiocardiography	BAS	Detected with angiocardiography during BAS
				Arterial switch procedure	
				Division of RAo	Good repair with no outflow tract obstruction. Normal growth with no evidence of stridor or esophageal dysfunction
				Closure of ASD and VSD	
				Ligation of PDA	
Tůma et al. (6)	1 day/boy	D-TGA, DAA, left-sided PDA, VSD, PFO	Angiocardiography	BAS	Detected with angiocardiography during BAS
				Senning operation	
				Division of LAo	Death from *Pseudomonas* pneumonia secondary to tracheobronchomalacia
				Ligation of PDA	
				Tracheostomy	
McMahon et al. (7)	1 day/girl	D-TGA, DAA, VSD, PDA (no side description), PFO	Intraoperative inspection	BAS	Found during surgery
				Arterial switch	Mild stridor in the postoperative period improved at the 12-month follow-up
				Division of RAo	
				Closure of ASD	
				Ligation of PDA	
Cui et al. (4)	2 days/boy	D-TGA, DAA, right-sided PDA, PFO	Echocardiography	Arterial switch	Detected with echocardiography
				Division of RAo	
					No respiratory distress
				Ligation of PDA	
Yao et al. (10)	Antenatal life/girl	D-TGA, DAA, PFO, no PDA description	Fetal echocardiography	Parents decided to terminate the pregnancy	Detected with fetal echocardiography
Anuwutnavin et al. (9)	Antenatal life	D-TGA, VSD, DAA, PS, and subpulmonary obstruction, no PDA described	Fetal echocardiography	BAS	Detected with fetal echocardiography.
				Blalock–Taussig shunt, division of RAo, aortopexy	
					Progressive desaturation
Dan Zhou and Laichun Song (14)	Postnatal diagnosis	D-TGA, VSD, PDA	Echocardiography CT scan	Arterial switch	The D-TGA detected with echocardiography and DAA found by CT scan after an arterial switch
					Correction of the DAA with good result
Our case (2023)	Antenatal life/boy	D-TGA, DAA, Left Tortuous PDA, VSD, PV bicuspid and dysplastic, Subpulmonary obstruction, PFO	Fetal echocardiography	BAS	Detected with a fetal echocardiography and confirmed with a postnatal echocardiography
				Division of RAo	
				Ligation PDA	
				Tracheopexy, aortopexy, arterial switch with VSD closure, and subpulmonary resection	An CT angiography evidenced tracheal compression and tracheobronchomalacia
					Progressive desaturation

ASD, atrial septal defect; BAS, balloon atrial septostomy; LAo, left aorta; PDA, patent ductus arteriosus; PFO, patent foramen ovale; PS, pulmonary stenosis, RAo right aorta.

The clinical presentation of patients with vascular rings is highly variable. Respiratory symptoms appear in 91% of presentations of the DAA, such as barky cough, wheezing, shortness of breath, and dysphagia, especially for solid food, with stridor being the most common presentation (77%) (11).

A multicenter study reported that respiratory signs were present in 21% of neonates on the day of birth: stridor was the most common clinical sign evaluated. Furthermore, 23% of the sample population were initially asymptomatic and not operated upon in the first year, and of these, 27% of patients developed respiratory symptoms/signs between 1 and 2 years of age (15).

Published data on the DAA indicate an approximately 5% association with array abnormalities. Postnatal series on the DAA are reported in the literature, and these describe an association of the DAA with 22q11 microdeletion. Other genetic anomalies that have been identified are trisomy 21, 15q24 microdeletion, and benign copy number variant (16). The majority of patients with a D-TGA have undefined genetics (17–19). The prevalence of genetic anomalies in patients with a D-TGA/VSD/PS and DAA has not been estimated; in the case of our patient with this combination, the results of the karyotype and array-CGH tests were negative. Screening test results for autoimmune and humoral immune function were also negative.

Fleenor et al. observed that patients with a VR who are symptomatic have a significantly altered tracheal geometry compared with non-symptomatic individuals in all dimensions measured, including the area and the longest and shortest diameters (20). In addition, tracheal compression impacts mucociliary clearance, leading to secretion stasis; symptoms become evident during expiration when the intrathoracic pressure (Ppl) overcomes the intratracheal pressure (Ptr), leading to a worsening of airway narrowing (20, 21). The worsening of symptoms is explained by Bernoulli's principle, according to which the increase in speed of a fluid occurs simultaneously with a decrease in pressure at the point where the channel narrows. This may result in inadequate removal of secretions (22). This, in turn, can lead to the recurrence and/or prolongation of respiratory infections.

Although a DAA carries a good prognosis, its association with tracheal anomalies could worsen the outcome (23). Reported that tracheal stenosis with pulmonary or cardiovascular malformations was associated with a 79% mortality rate.

The gold standard of treatment is surgical correction. Furthermore, an accurate estimation of the degree of tracheal compression during the prenatal period is a challenging task, yet it significantly impacts patient outcomes. In the case of our patient, it could be assumed that, although there is no evidence of tracheal compression given the rarity of the condition, the unpredictability of tracheal compression is also related to the different anatomical arrangements of the great vessels in the D-TGA. In fact, in addition to the compression and the tracheomalacia determined by the VR, the anterior position of the aorta and its course could contribute to a worsening of compression. Furthermore, the posterior pulmonary artery could cause a displacement of the trachea and its bifurcation, influencing respiratory dynamics. This may predispose to nosocomial infections along with a prolonged hospitalization in the ICU.

In conclusion, a D-TGA with a DAA remains an extremely unusual association. The outcome of patients with this condition presents a high degree of variability and is entirely unpredictable in prenatal life. Factors influencing this phenotypic range are related not only to intracardiac anatomy but also to the arrangement of the great vessels, tracheal compression, and tracheomalacia associated with the VR. The last two features represent negative prognostic factors, as they determine an increased risk of prolonged ICU stay and prolonged intubation.

Our greatest aim as fetal and perinatal cardiologists is to improve the management and outcome of patients with the above condition through a fetal diagnosis, recognizing types of congenital heart disease in newborns who require early neonatal invasive procedures.

## Data Availability

The original contributions presented in the study are included in the article/Supplementary Material, further inquiries can be directed to the corresponding author.
